# Altered Cortico–Striatal Functional Connectivity During Resting State in Obsessive–Compulsive Disorder

**DOI:** 10.3389/fpsyt.2019.00319

**Published:** 2019-05-10

**Authors:** Jessica Calzà, Deniz A. Gürsel, Benita Schmitz-Koep, Benno Bremer, Lena Reinholz, Götz Berberich, Kathrin Koch

**Affiliations:** ^1^Department of Neuroradiology, Technical University of Munich, School of Medicine, Klinikum rechts der Isar, Munich, Germany; ^2^TUM-Neuroimaging Center (TUM-NIC) of Klinikum rechts der Isar, Technische Universität München (TUM), Munich, Germany; ^3^Department of Psychology, Ludwigs-Maximilians-Universität, Munich, Germany; ^4^Windach Institute and Hospital of Neurobehavioural Research and Therapy (WINTR), Windach, Germany; ^5^Graduate School of Systemic Neurosciences GSN, Ludwig-Maximilians-Universität, Biocenter, Munich, Germany

**Keywords:** obsessive–compulsive disorder, cortico–striato–thalamo–cortical, fronto–striatal, connectivity, resting-state, subthalamic nucleus

## Abstract

**Background:** Neuroimaging studies show that obsessive–compulsive disorder (OCD) is characterized by an alteration of the cortico–striato–thalamo–cortical (CSTC) system in terms of an imbalance of activity between the direct and the indirect loop of the CSTC. As resting-state functional connectivity (FC) studies investigated only specific parts of the CSTC in patients with OCD up to now, the present study aimed at exploring FC in the CSTC as a whole.

**Methods:** We investigated potential alterations in resting-state FC within the CSTC system in 44 OCD patients and 40 healthy controls by taking into consideration all relevant nodes of the direct and indirect CSTC loop.

**Results:** Compared to healthy controls, OCD patients showed an increased FC between the left subthalamic nucleus (STN) and the left external globus pallidus (GPe), as well as an increased FC between the left GPe and the left internal globus pallidus (GPi).

**Conclusion:** These findings may contribute to a better understanding of the OCD pathophysiology by providing further information on the connectivity alterations within specific regions of the CSTC system. In particular, increased FC between the STN and the left GPe may play a major role in OCD pathology. This assumption is consistent with the fact that these regions are also the main target sites of therapeutic deep brain stimulation in OCD.

## Introduction

Obsessive–compulsive disorder (OCD) is a psychiatric disease with a lifetime prevalence of 2–3%. It is characterized by two main symptoms, obsessions and compulsions. Obsessions are described as recurrent and persistent thoughts or impulses perceived as unwanted and intrusive, for instance, the fear of contamination, the fear of causing harm to the self or to other people, or obsession for symmetry. Compulsions are defined as repetitive behaviors or mental thoughts aiming at reducing distress and anxiety, such as washing, checking, or counting.

According to the traditional neurobiological model, OCD is characterized by an aberrant activity of the cortico–striato–thalamo–cortical pathway (CSTC). The CSTC includes the orbitofrontal cortex (OFC), the anterior cingulate cortex (ACC), the basal ganglia, and the thalamus (1, 2). These regions have also repeatedly been reported to be affected by structural alterations, with regard to both white and gray matter abnormalities (3, 4). Predominantly, systematic meta-analyses isolated alterations in fronto-basal white matter pathways targeting the OFC and the ACC (4, 5), indicating that microstructural changes in long-range connections within the CSTC might constitute the basis of the aberrant functional activity of this system.

It is possible to distinguish two main pathways that characterize the CSTC system, the direct and the indirect loop. The direct loop refers to the projections from the cortex to the striatum to the internal globus pallidus and pars reticulata of substantia nigra (GPi/SNr). From the GPi/SNr, the direct loop projects then to the thalamus, which, finally, projects back to the cortex. In total, the direct loop has two inhibitory projections and two excitatory projections, and it finally results in an activation of the cortex. For this reason, it is defined as a positive-feedback loop (2).

The indirect loop, instead, includes projections from the cortex to the striatum to the external globus pallidus (GPe) and then to the subthalamic nucleus (STN). From the STN, it projects to the GPi/SNr, reconnecting to the direct loop, which, in turn, projects to the thalamus and finally back to the cortex. The indirect pathway involves three excitatory and four inhibitory projections; thus, it is thought to have an inhibitory effect on the cortex, which makes it a negative-feedback loop (2).

The CSTC is involved in various cognitive and emotional processes such as reward-based learning, decision making, and goal-direct behavior in response to significant stimuli (6, 7). But most of all, it is known to be involved in motor functions, such as procedural and habit learning, appropriate action selection and execution, action inhibition, and control of impulsivity (2, 8). Specifically, the direct loop is thought to excite the cortex with the result of action execution. The indirect loop, instead, inhibits the direct loop with the consequential stop of impulsive behavior and inhibition of actions that are no more relevant or no more adequate to the situation (8). Hence, the balance between direct and indirect loop activity is pivotal for a correct motor behavior and a correct selection of adaptive actions.

Neuroimaging studies suggest that obsessive–compulsive disorder is characterized by an altered activation in the cortico-striatal circuitry in terms of an overall hyperactivity of the cortico-striatal loop.

One hypothesis widely accepted is that the hyperactivation of the CSTC is due to an imbalanced activation of the direct and the indirect loop (2, 8). According to this hypothesis, OCD patients are affected by an excessive activation of the excitatory “positive-feedback” loop (direct loop). In consequence, the indirect loop is no longer able to regulate the activity of the direct pathway and the cortex. This results in a cortical hyperactivation leading to the typical symptoms of OCD, e.g., impulsivity and impaired action inhibition.

Resting-state functional connectivity (FC), defined as the temporal correlation of neuronal activation between different brain regions during rest, has been used to investigate alterations of connectivity within the CSTC system in OCD. Using this approach, some studies found an increased FC within some structures in the direct loop of the CSTC, such as the cortex, the striatum, and the thalamus, in OCD patients during resting state (6, 9–12). These findings might support the prevailing neurobiological hypothesis of a hyperactivation of the direct loop. For instance, Fitzgerald et al. (6) reported an increased FC between the dorsal striatum and the frontal pole during resting-state fMRI, and similar results were observed between the ventral caudate/nucleus accumbens and the OFC and ACC by Harrison et al. (9). Yet, Jung et al. (12) reported a decreased FC between OFC and striatum and an increased connectivity between striatal areas and thalamus. Evidence shows that the results of FC within the CSTC are heterogeneous and somehow controversial. Indeed, there are studies that showed different results, as, for instance, Chen et al. (13), who found a general decrease of FC within the CSTC during resting state, or Beucke et al. (14), who reported an increased FC between the OFC and the STN, which is the main output structure of the indirect loop of the CSTC. This result heterogeneity may be partly due to methodological differences between the studies. Thus, whereas some used data-driven, model-free methods to investigate whole-brain FC during rest (e.g., 13–17), others investigated seed-based FC predominantly without direct relation to the CSTC [for a review, see Ref. (18)] or FC of selected CSTC nodes [e.g., Refs. (6, 9, 12)]. While findings of these studies can still be assumed to make a considerable contribution to a better understanding of the mechanisms underlying connectivity alterations in OCD, none of these studies investigated connectivity within the CSTC as a whole, i.e., between all nodes known to constitute anatomically relevant relay stations of the direct and indirect CSTC loop (1, 2, 19–21).

Against this background, using resting-state functional magnetic resonance imaging (fMRI), the present study investigated Region of interest ROI-to-ROI connectivity within all nodes of the direct and indirect CSTC loop in a relatively large sample of OCD patients and healthy controls to improve our understanding of how the CSTC system contributes to OCD pathology.

## Methods

### Participants

Forty-four adults with OCD (14 males, 30 females) and 40 healthy controls (19 males, 21 females) took part in this study. Patients were recruited from Windach Institute and Hospital of Neurobehavioural Research and Therapy (WINTR), Germany, where the assessment of the disorder was performed by an experienced psychiatrist based on the criteria of Diagnostic and statistical manual of mental disorders (DSM-5) for OCD diagnosis. In addition, we used the Yale-Brown Obsessive Compulsive Scale (Y-BOCS) to evaluate the severity of OCD symptoms in patients before the scanning procedure and the Obsessive Compulsive Inventory-Revised (OCI-R) to assess the main OCD symptom dimensions. Hamilton Depression Scale was performed to assess the presence of depression. Healthy controls with a history of psychiatric illness were excluded. Exclusion criteria for both groups were a history of clinically important head injuries, seizures, neurological diseases, schizophrenia, autism, substance and alcohol abuse/dependency, mental retardation, pregnancy, and severe medical conditions. At the time of the scanning, 17 patients were under medication, while the other 27 were medication-naïve or stopped medication at least 1 week before scanning (see **Table 1**).

**Table 1:** Demographic and clinical data.

	Patients	Controls	Group difference
**Number**	44	40	
**Gender**	14 males 30 females	19 males 21 females	n.s.
**Mean age**	33.32 (SD = 11.35)	34.12 (SD = 8.81)	n.s.
**Duration of OCD (in years)**	14.93 (SD = 11.84)	−	
**Age of onset**	17.34 (SD = 8.14)	−	
**Y-BOCS mean total score**	21.41 (SD = 5.94)	−	
Y-BOCS Obsessions Y-BOCS Compulsions	10.88 (SD = 3.42) 10.52 (SD = 3.84)	− −	
**OCI-R mean total score** OCI-R washing OCI-R checking OCI-R neutralizing OCI-R obsessing OCI-R ordering OCI-R hoarding	27.73 (SD = 10.15) 5.00 (SD = 4.25) 5.34 (SD = 3.48) 2.70 (SD = 3.27) 7.43 (SD = 3.40) 4.45 (SD = 3.63) 2.80 (SD = 2.72)	− − − − − − −	
**Hamilton Depression Scale (HAMD) mean score**	13.14 (SD = 5.56)	0.70 (SD = 1.08)	
**Medication** SSRI SSRNI Methylphenidate Neuroleptic TCA	17 15 2 1 1 2	−	
**Comorbidity** Depression Anxiety disorder attention deficit hyperactivity disorder (ADHD) Personality disorder	19 12 5 2 1	−	

Multiple medications and comorbidities can be present in a single subject.

SSRI, selective serotonin reuptake inhibitor; SSNRI, selective serotonin-norepinephrine reuptake inhibitor; TCA, tricyclic antidepressant; OCD, obsessive–compulsive disorder; Y-BOCS, Yale-Brown Obsessive Compulsive Scale; OCI-R, Obsessive Compulsive Inventory-Revised.

After a detailed description of the study, patients gave their written informed consent to participate. The present study was approved by the Ethics Committee of the Klinikum rechts der Isar in München, and it was in accordance with the Declaration of Helsinki.

### Image Acquisition

The resting-state fMRI images were collected on a Philips Ingenia 3.0 T whole body system equipped with a 32-channel head coil. Resting-state fMRI data were acquired using a gradient echo-planar (EPI) sequence, with the following parameters: The functional MRI resting-state data were acquired with a gradient echo EPI sequence: scan duration = 551 s, echo time (TE) 33 ms, repetition time (TR) = 2,700 ms, flip angle 90°, field of view (FOV) = 192 × 192 × 141 mm^2^, matrix = 96 × 94, 64 slices, transverse orientation, slice thickness = 2 mm, and 0.2-mm interslice gap, number of volumes = 200. During rest, participants were instructed to keep their eyes closed, relax, and try to avoid falling asleep. High-resolution structural images were acquired using a magnetization-prepared rapid acquisition gradient echo (MPRAGE) sequence (TE = 5.09 ms, FA = 8°, FOV = 239 × 256 × 161 mm, pixel matrix = 384 × 384, scan duration = 299 s, slice thickness = 0.7 mm).

### Statistical Analysis

Imaging data were preprocessed using the default preprocessing parameters of CONN Functional Connectivity toolbox conn v.17.f for Statistical Parametric Mapping (https://www.nitrc.org/projects/conn). Slice timing correction based on slice order and subject motion correction were performed. All data were inspected for movement artifacts. Subjects with movement parameters exceeding 3 mm of translation on the x-, y-, or z-axis or 3° of rotation were excluded (n = 0). In addition, excessive head motion was established with framewise displacement (FD), calculated as the sum of the absolute values of the derivatives of the six motion parameters derived from SPM12 (22). No significant differences in mean FD (p = 0.83) were found between healthy controls (mean = 0.11, sd = 0.03) and OCD patients (mean = 0.11, sd = 0.04). Data were then normalized to a standard template in Montreal Neurological Institute space and smoothed with an 8-mm Gaussian smoothing kernel. ART software procedure was applied to detect and regress out possible outliers and artefacts due to head movements. A band-pass filter (0.008–0.09) was applied. Confounding white matter and cerebrospinal fluid components, as well as the six head motion parameters generated during realignment process, were entered at a first-level correlation analysis as nuisance covariates.

For each participant, bivariate correlation analysis was computed between each pair of ROIs (ROI-to-ROI analysis), using time series information. Correlation matrices of each participant were then Fisher z-transformed and entered in a second-level analysis. A two-sample t-test, corrected for age and gender, was performed in order to estimate differences in FC between healthy participants and patients with OCD. In addition, we performed a correlation between ROI-to-ROI FC measures and OCD symptom severity (as assessed by the Y-BOCS total, obsession, and compulsion scores) as well as medication in OCD patients. To do so, we extracted the FC beta values of the ROIs that showed significant difference in FC between groups and correlated them with the measures of interest, i.e., Y-BOCS total scores, Y-BOCS obsession scores, Y-BOCS compulsion scores, and medication. Medication was entered as a covariate, indicating the state of medication in terms of presence or absence in patients. Patients were considered not medicated only if they were medication-naïve or stopped the medication at least 1 week before the scanning.

**Figure 1:**
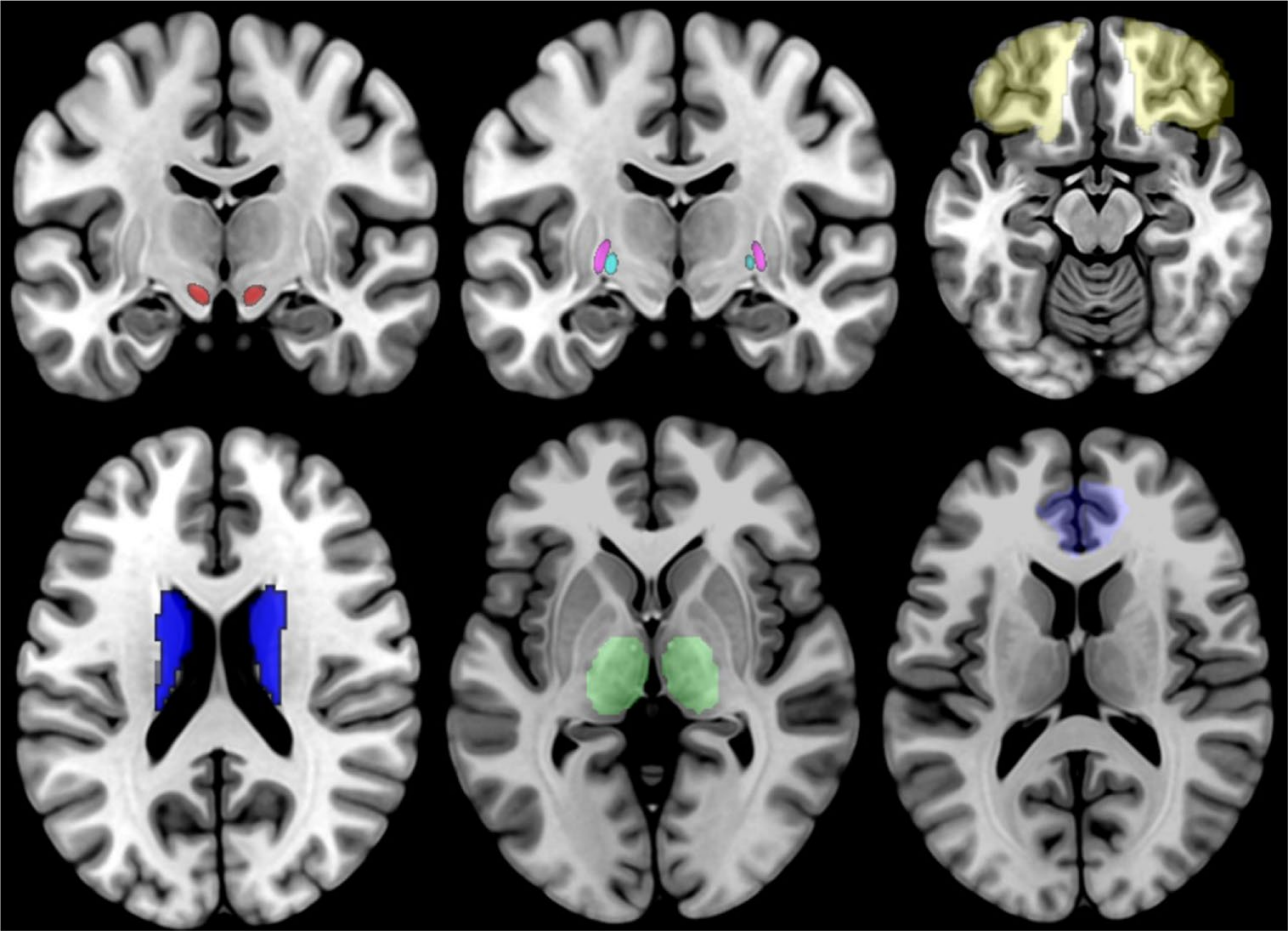
Illustration of the 14 ROIs, with the STN shown in red, the GPi shown in cyan, the GPi shown in violet, the OFC shown in yellow, the striatum shown in blue, the thalamus shown in green, and the ACC shown in light blue. STN, subthalamic nucleus; GPi, internal globus pallidus; OFC, orbitofrontal cortex; ACC, anterior cingulate cortex.

For our analyses, we selected 7 bilateral seeds, for a total of 14 seeds, which correspond to the structures that are part of the direct and indirect CSTC loop (21). These ROIs are the OFC, the ACC, the striatum, the STN, the internal and external globus pallidus (GPi and GPe, respectively), and the thalamus (see **Figure 1**). The GP and STN ROIs were selected among the ROIs provided by the 7T ATAG atlas of Basal Ganglia, while all the other seeds corresponded to the ROIs provided by the AAL2 atlas. For the ROI-to-ROI analysis, we looked at bidirectional FC results and considered significant only those regions that survived at a peak level threshold of p < 0.05 corrected for multiple comparisons [false discovery rate (FDR)] in both directions.

## Results

### ROI-to-ROI Analysis

**Figure 2:**
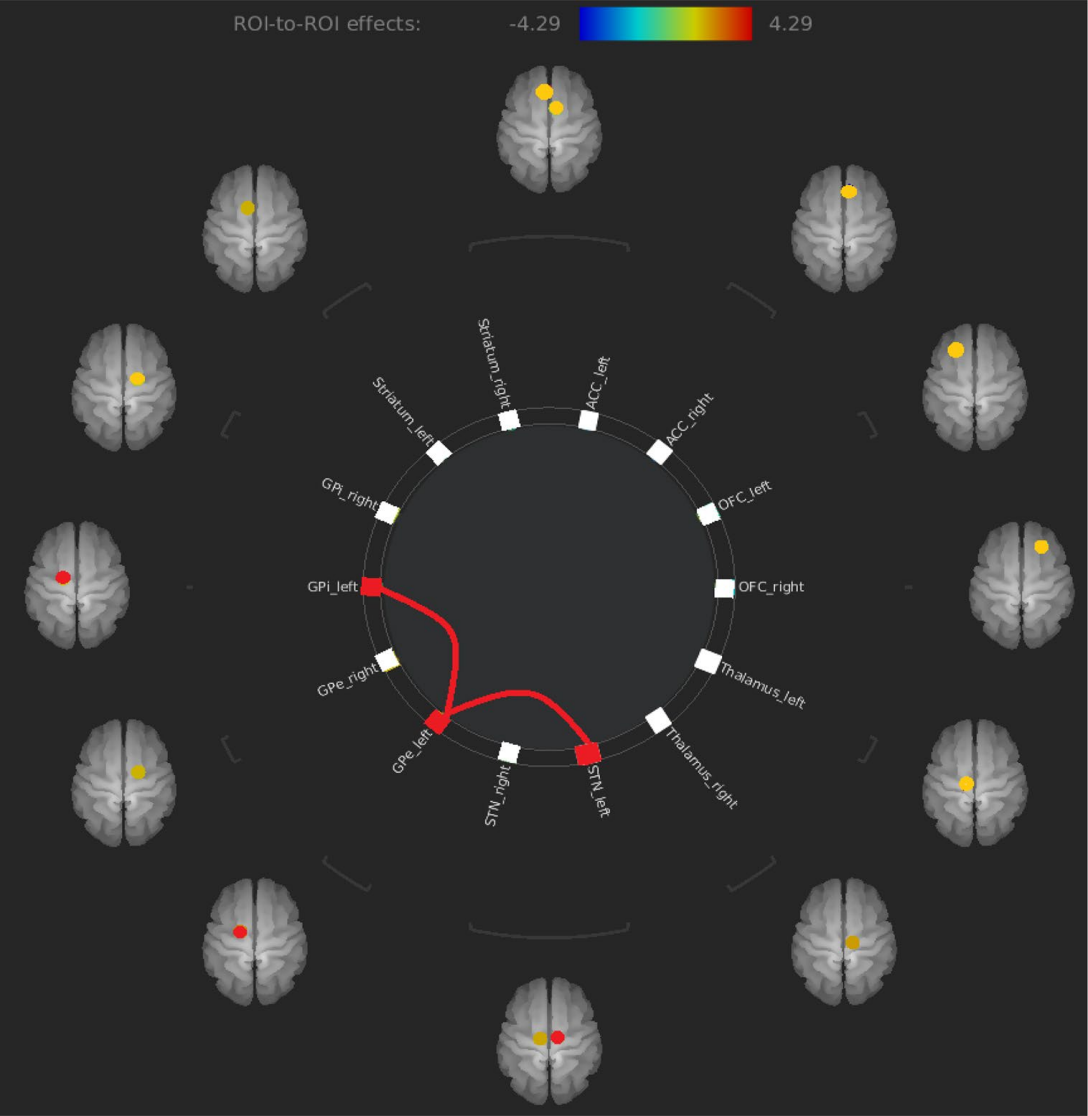
Increased FC between left STN and left GPe and increased FC between left GPe and left GPi in OCD patients compared to healthy controls. FC, functional connectivity; GPe, external globus pallidus; OCD, obsessive–compulsive disorder.

The only results that survived at an FDR corrected threshold corresponded to an increased FC between the left STN and the left GPe (t = 4.29) and an increased FC between the left GPe and the left GPi (t = 3.66) in OCD patients compared to healthy controls (see **Figure 2** and **Table 2**).

**Table 2:** Significant results of ROI-to-ROI analysis.

ROI-to-ROI	T-value	p-value (FDR corrected)
Left STN - Left GPe	T = 4.29	0.0046
Left GPe - Left GPi	T = 3.66	0.0203

### Correlation With Symptom Severity

Correlation between FC measures and OCD symptom severity (Y-BOCS total, Y-BOCS obsessions, Y-BOCS compulsions) did not yield any significant results.

### Correlation With Medication

Likewise, correlation between FC measures and medication did not yield any significant results.

## Discussion

In the present study, we investigated potential alterations of FC within the CSTC system during resting state in patients with OCD by taking into consideration all relevant nodes of the direct and indirect CSTC loop. We found an increased FC between the left STN and the left pars externa of the GP, as well as an increased FC between the left internal and left external GP in OCD patients compared to healthy controls.

Comprehensive meta-analyses exploring functional or gray/white matter structural alterations in OCD reported changes partly in regions of the CSTC, such as the pallidum (23), but also in areas and networks not directly pertaining to the CSTC, such as dorsomedial, dorsolateral, ventrolateral, and frontopolar prefrontal cortices (3), temporal and parietal regions (3, 24), or—regarding functional alterations—the default mode network (18).

Nevertheless, alterations within the classic CSTC loop are still regarded as a central psychopathological mechanism in OCD. Our findings support this prevailing neurobiological model of OCD stating an alteration of the CSTC system.

The predominant hypothesis of this model assumes that this alteration corresponds to an increased FC within the direct pathway of the CSTC, specifically between the cortex and the striatum, the striatum and the GPi/SNr, and the thalamus and the cortex. However, results from different studies on CSTC FC in OCD are controversial and, at the best of our knowledge, there are not many studies that clearly corroborate this hypothesis.

The present study introduces a new perspective on CSTC function in OCD. Representing the first study of FC in all relevant nodes of the direct and indirect CSTC loop, its findings add to the few existing evidence showing an increased FC between prominent output structures of the indirect pathway of the CSTC. Although our results seem to contrast the prevailing view of the model, they are very plausible under the following considerations.

First, increased FC between the external and internal GP is consistent with the hypothesis that the CSTC model and the functional and structural connectivity between the regions that are part of it are more complex than initially thought. In particular, different animal studies report a direct structural connection between the external and the internal globus pallidus, suggesting that the role of the GPe is more central and complex as stated by the traditional CSTC model, and it probably has a role in controlling the function of the GPi/SNr (19, 25, 26). Our findings of increased FC between external and internal GP might support this hypothesis.

Accordingly, the increased FC between STN and GPe might be in accordance with the hypothesis of a major central role of GPe in the CSTC system and might indicate that that STN and GPe play a major role in OCD pathophysiology. More precisely, the altered FC between the STN and the GPe may disrupt the balance between the direct and indirect loops with a consequent alteration in cortex excitation, leading, in turn, to the characteristic cognitive and motor symptoms of OCD. In line with this assumption, the STN is a region that is currently receiving increasing attention regarding OCD pathology. It is considered to constitute the main output structure of the basal ganglia given its efferent projections through which it modulates the activation of the GPi/SNr and, finally, the cortex. It is involved in different cognitive, affective, and motor functions, such as decision making, reward processing, emotional evaluation of external stimuli (27), and most of all control of impulsivity and action inhibition (28–31). Recent findings demonstrated an alteration of FC involving the STN in patients with OCD. For instance, Beucke et al. (14) found that patients with OCD had significantly greater distant connectivity (defined as correlation to voxels exclusively outside a 12-mm sphere) in the STN compared to healthy controls. Morrie et al. (32) found a correlation between decreased FC of the STN with the premotor cortex and perseveration traits and between decreased FC of the STN with the dorsolateral prefrontal cortex (DLPFC) and higher obsessive–compulsive traits. Recently, Cano et al. (33) showed altered FC involving the STN during resting state in OCD patients. All these studies are in line with our findings and support the hypothesis that the STN might be a key structure in OCD pathology.

In concordance with this hypothesis, the STN is often used as a target region for deep brain stimulation (DBS) in OCD. DBS is a neurosurgical treatment used for treatment refractory OCD, which represents 10–20% of all cases. It consists of an implantation of electrodes in specific brain regions that are then electrically stimulated. This stimulation results in a sustained improvement of OCD symptoms in the majority of the patients under treatment (34–39). The mechanisms underlying DBS are still unknown, but it is thought to restore the balance between the activity of the direct and indirect loops within the CSTC (40, 41). Different studies of DBS in the STN show that the stimulation of this area leads to an improvement of previously treatment-refractory OCD symptoms, defined by a decrease in Y-BOCS total score, that can vary between 50% and 75% (29, 34, 41, 42). Our results could represent a neurobiological framework that allows us to interpret the mechanism underlying DBS treatment. In particular, we believe that the increased FC between the STN and GPe reflects an increased concerted activity of these areas going along with a disrupted balance within the CSTC. STN-DBS has been shown to exert an inhibitory effect both on the target area and on distal structures, such as the GPe and GPi, thus decreasing elevated activity of these structures and leading to an improvement in OCD symptoms. This is also supported by animal studies showing that high-frequency stimulation (HFS), as well as pharmacological inhibition of STN, reduces compulsive symptoms induced by the administration of quinpirole in rats (43). The effect of the STN-DBS on distant structures, such as the GPi and GPe, is demonstrated by human studies showing simultaneous local and distal effects of electrical stimulation of specific brain regions (44–46). In light of these considerations, it might seem surprising that we did not find an association between altered FC and symptoms. However, although the altered FC does not seem to be directly related to the degree or severity of clinical symptoms, the alteration may still represent a core psychopathological mechanism which—although not directly correlated with the degree of clinical severity—might to some degree normalize under DBS treatment.

## Limitations

Finally, we must highlight some limitations. One limitation is represented by the fact that, although OCD was the primary diagnosis in all patients, some patients suffered from comorbidities, such as depression and anxiety disorder, that may have affected our results to some degree. Second, some patients were medicated at the time of scanning. Although we found no effect of medication in our analyses, a certain influence of selective serotonin reuptake inhibitor (SSRI) medication on FC cannot fully be excluded given studies showing that both functional and structural abnormalities are responsive to SSRIs (47, 48). SSRIs are considered to have a neuroprotective effect and to promote brain-derived neurotrophic factor (BDNF) activation, thus directly increasing central nervous system myelination (49). It has even been suggested that the clinical efficacy of SSRIs may be related to a post-treatment normalization of FC in fronto–striatal networks and/or thalamo–cortical pathways connected to posterior brain regions (4). Hence, against the background of these findings and assumptions, the fact that the majority of the patients in our sample were receiving antidepressant treatment must—despite the lack of a statistically significant effect—be regarded as a major limitation.

## Conclusion

The present study investigated FC within the CSTC in patients with OCD. We found an increased FC between the left STN and the left GPe, as well as an increased FC between the left GPe and the left GPi. These results point to an alteration of FC between central nodes of the indirect loop of the CSTC and suggest that STN might play a major role in OCD pathophysiology. Moreover, results of the present study may explain the general mechanism underlying STN-DBS efficacy.

## Ethics Statement

The study has been approved by the Ethics Committee Klinikum rechts der Isar.

## Author Contributions

KK and GB designed the study and wrote the protocol. JC managed the literature searches and analyses. JC undertook the statistical analysis and wrote the first draft of the manuscript. DAG, BS-K, BB, and LR recruited and screened the patients. GB, DAG, BS-K, BB, LR, and KK added additional aspects to the final manuscript. All authors have approved the final article.

## Funding

This study was supported by a Deutsche Forschungsgemeinschaft (DFG) grant to KK (KO 3744/7-1).

## Conflict of Interest Statement

The authors declare that the research was conducted in the absence of any commercial or financial relationships that could be construed as a potential conflict of interest.
